# *In vitro* ovicidal studies on egg-parasitic fungus *Pochonia chlamydosporia* and safety tests on mice

**DOI:** 10.3389/fvets.2024.1505824

**Published:** 2025-01-09

**Authors:** Yuan Ma, Jinbao Lv, Lili Jiang, Zhaobin Fan, Luyao Hao, Zhengyi Li, Chengyu Ma, Rui Wang, Hongliang Luo

**Affiliations:** ^1^College of Veterinary Medicine, Inner Mongolia Agricultural University, Hohhot, China; ^2^Key Laboratory of Clinical Diagnosis and Treatment of Animal Diseases, Ministry of Agriculture, National Animal Medicine Experimental Teaching Center, Hohhot, China; ^3^National Center of Technology Innovation for Dairy, Hohhot, China; ^4^Zhongnong Dong Jun Animal Diagnosis Technology (Beijing) Co., Ltd., Beijing, China; ^5^College of Pharmacy Heze University, Heze, China; ^6^Rui Pu Agricultural Technology Co., Ltd., Hohhot, China

**Keywords:** biocontrol, *P. chlamydosporia*, eggs, toxicity, mice, parasites

## Abstract

**Introduction:**

The control of parasites infections in livestock is an ongoing concern, with parasites developing resistance to commonly used antiparasitic drugs. The current study investigated *in vitro* the destructive effect of the fungus Pochonia chlamydosporia on the eggs and oocysts of several equine parasites, as well as assessing the safety of the fungus in mice.

**Methods:**

*S. equinus*, *P. equorum*, Anoplocephala spp eggs and *Eimeria* spp. oocysts were treated with *P. chlamydosporia*. The prepared preparation was also administered to mice, and the physiological indexes and lesions of major tissues and organs, as well as pathological sections of tissue, were then observed.

**Results:**

*P. chlamydosporia* exhibited varying degrees of efficacy in the control of *S. equinus*, *P. equorum*, *Anoplocephala* spp eggs and *Eimeria* spp. oocysts. The acute toxicity test demonstrated that there was no death or toxicity symptom observed in the mice, with no significant difference in clinical observations, such as respiration, mental state, appetite, or feces, between the control and treated mice after the feeding of the biological preparation of *P. chlamydosporia*.

**Discussion:**

These findings suggested that administration of *P. chlamydosporia* would be safe to use in livestock and provided a rationale for its potential clinical application, pending further analyses.

## 1 Introduction

Anthelmintic drugs are widely used to control parasitic diseases of livestock, which can otherwise result in high economic losses as a result of significant morbidity. However, the long-term use of such drugs can result in the development of resistance, rendering them increasingly less effective (1–3). Therefore, researchers have begun to investigate the use of natural enemies for the biological control of parasites (4). Such an approach also helps improve animal food safety, environmental protection, and the sustainable development of animal husbandry.

Nematophagous fungi, including Zygomycotina, Deuteromycotina, Basidomycotina, and Ascomycotina, are natural enemies of parasites and, thus, have an important role in the biological control of both plant and animal parasites (5). They can be classified into four major groups based on the mode of nematode attack: (i) nematode-trapping/predators, (ii) opportunistic or ovicidal, (iii) endoparasites, (iv) toxin-producing fungi and (v) producers of special attack devices (6). They can reproduce in three ways, via aerial mycelia, conidia, and chlamydospores, and spread primarily by mycelial breakage and conidial germination. Fungal conidia are ingested by animals and excreted in the feces, where they can germinate to then kill any parasitic eggs; thus, conidia can be used as food additives for livestock to prevent and control parasitic eggs.

*Pochonia chlamydosporia* (38) is a ovicidal fungus of the Hypocreales (Clavicipitaceae) belonging to Ascomycota. *Pochonia chlamydosporia* has shown efficacy *in vitro* and *in vivo* for the biocontrol of gastrointestinal parasites in domestic animals by parasitizing eggs and female nematodes (7), while *Monacrosporium sinense* [belongs to *Arthrobotrys*, (39)] captures nematode larvae via adhesive networks (8). The combination of *M. sinense* and *P. chlamydosporia* demonstrated a 98.90% reduction in the number of bovine nematode infective larvae under *in vitro* conditions at 25°C (9). In a study by Braga et al. (10), *P. chlamydosporia* (VC4) was successfully administered orally to horses in the form of pellets. Although available data indicate that *P. chlamydosporia* kills the eggs of a variety of parasites, there is no detailed report on which eggs of equine parasites are affected and how effectively, or whether the fungus is safe for use in animals. Thus, the current study investigated the impact of *P. chlamydosporia* on the eggs and oocysts of equine parasites and examined the safety of a lyophilized preparation in mice to explore the possibility of its application for the control of parasites. The results lay the foundation for the clinical application of *P. chlamydosporia* as an effective and sustainable biological control method for the prevention and control of animal parasitic diseases.

## 2 Materials and methods

### 2.1 Materials

#### 2.1.1 Test strains, eggs, and oocysts

A test strain of *P. chlamydosporia*, ARSEF3539, was obtained from the Veterinary Parasite Laboratory of the Inner Mongolia Agricultural University, Hohhot, Inner Mongolia, China.

A floating method was used for the separation of *Strongylus equinus*, *Parascaris equorum*, *Anoplocephala* spp. eggs and *Eimeria* spp. oocysts from feces excreted by naturally infected horses. Light microscopy (20×) was used for the morphological analysis of the eggs and oocysts. Eggs and oocysts were collected and washed with distilled water, 3,000 g, for 5 min. They were then sterilized using a 1% NaClO solution and their morphological integrity was analyzed under an optical microscope. Centrifugation was repeated and the intact eggs and oocysts were washed a further three to five times to remove the NaClO solution. A suspension of 1,000 eggs or oocysts/mL was then prepared.

#### 2.1.2 Preparation of culture medium and solution

Potato dextrose agar (PDA) medium (HopeBio) was prepared using water agar (WA) medium, whereby 15 g of agar was added to 1 L of water and mixed, before heating to 121°C for 15 min. Spore eluate was prepared by adding 1 mL Tween-80 to 500 mL of water, and then heating it to 121°C for 15 min.

#### 2.1.3 Preparation of lyophilized biologics

**Figure 1:**
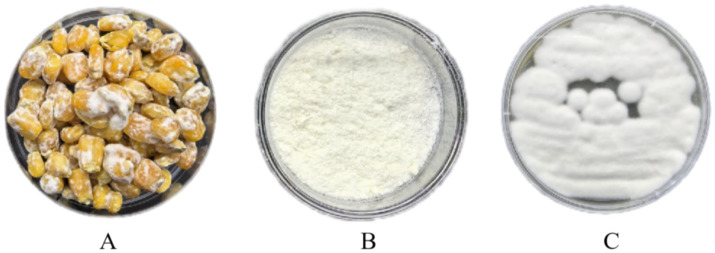
Lyophilized biologics of *Pochonia chlamydosporia*. **(A)** *P. chlamydosporia* culture; **(B)** *P. chlamydosporia* lyophilized powder; **(C)** *P. chlamydosporia* powder culture.

*Pochonia chlamydosporia* was inoculated into PDA medium until the mycelium covered the surface of the petri dish; this was then cut into 0.5 cm × 0.5 cm squares, and transferred to corn medium for 3 weeks (Figure 1A). The conidia were eluted from the medium with the spore eluate and filtered. The samples were prefrozen for 2 h at −80°C, and then placed in a vacuum freeze dryer. When the samples were dry powder, the conidia were counted (Figure 1B); the chlamydospore count in the lyophilized formulation was recorded as 2.13 × 10^8^ chlamydospores/g (Figure 1C). The dishes were then sealed with sealing film, and stored at 4°C.

### 2.2 Methods

#### 2.2.1 Impact of *P. chlamydosporia* on eggs and oocysts

The conidial suspension was spread evenly on WA medium spread on a dialysis membrane and left to grow mycelium for 3 days. Then 100 μL of each egg or oocyst suspension was added to different WA medium inoculated with *P. chlamydosporia*, with another 100 μL of each eggs or oocysts suspension being added to WA medium not inoculated with *P. chlamydosporia* as a control. Each petri dish was stored at 25°C for 7 days, with 10 repetitions for each treatment. After 7 days, the eggs or oocysts were photographed under a light microscope and examined for any signs of infestation; the integrity of the eggs or oocysts was evaluated according to the parameters established by Lýsek (11).

Equation 1 was used to obtain the percentage reduction in the number of eggs or oocysts after treatment (12):


(1) $$\begin{array}{l} \mathrm{Reduction}\%=(\mathrm{average number of eggs or oocysts}\\ \mathrm{in the control group}-\mathrm{average number of eggs or}\\ \mathrm{oocysts in the treated group})/(\mathrm{average number of}\;\\ \mathrm{eggs or oocysts in the control group})\times 100 \end{array}$$

#### 2.2.2 Acute transoral toxicity test in mice

The safety of the *P. chlamydosporia* inoculum was tested following the procedure of the Chinese National Food Safety Standard Acute Oral Toxicity Test (GB 15193.3–2014). Twenty male and 20 female mice, 6 ~ 8-weeks-old, and each weighing ~20 g were fasted for 6 h before use in the study. The recommended therapeutic dose of *P. chlamydosporia* was 1 × 10^6^ conidium/kg body weight (b.w.) (13). The mice were randomly divided into four groups: (1) normal dose group (1 × 10^6^ conidium/kg b.w.); (ii) 5 × dose group (5 × 10^6^ conidium/kg b.w.); (iii) 10 × dose group (1 × 10^7^conidium/kg b.w.); and (iv) control group, with 10 mice in each group. The lyophilized preparation of *P. chlamydosporia* was dissolved in distilled water and the appropriate dose was weighed according to the b.w. and administered orally to ensure that the preparation entered the stomach via the mouth. The control group was given the same stimulus by administering distilled water. Mice were fasted for 2 h after gavage, then housed normally, and observed continuously for 14 days.

During the test, mice were observed in terms of their mental status. Defecation was observed to see whether the mice had constipation or diarrhea. During the test period, all mice were uniformly fed with rat food 3 times/day. All mice in each group were weighed to determine the effect of the oral biologics on their b.w. Their body temperature was also measured before and 3, 7, and 14 days after inoculation. Blood was collected from each mouse on Day 14 after administration for routine blood and biochemical index examinations detected. At the end of the experiment, all mice were euthanized and the heart, liver, spleen, lungs, and kidneys were removed and weighed, and the organ coefficient was calculated using Equation 2:


(2) $$\mathrm{Organ coefficient}=\mathrm{organ weight}\;\left(\mathrm{mg}\right)/\mathrm{body weight}\;\left(g\right)$$

Samples of each tissue were taken for fixation, sectioning, and microscopic examination to observe their morphology and color.

#### 2.2.3 Mouse skin sensitization test

Eight healthy mice, four males and four females, were selected, and the hair on both sides of the spine was shaved off from an area of ~2 cm^2^ 24 h before the test so that the shaved area was determined to be free of abnormalities for 24 h before testing. Next, 0.5 mL of the *P. chlamydosporia* inoculum at a concentration of 1 × 10^8^/kg was applied to one of the shaved areas on each mouse, which were then covered for 4 h, after which the inoculum was removed. The other patch of shaved skin was used as a control. The intensity of sensitization was judged by comparing the erythema, swelling, and hardness of the local skin according at 24, 48, and 72 h.

#### 2.2.4 Acute eye stimulation test in mice

Eight healthy mice, four males and four females, were selected, and both eyes were examined 24 h before the test. No obvious sign of eye irritation, corneal defects, or conjunctival damage was observed. The animals were divided into a control group and a fungal stimulation group; the latter was treated once by taking 0.1 mL of inoculum (at a concentration of 1 × 10^8^ CFU/kg) and placing it directly into the left eye of the animal. The right eye of each of the test animals comprised the control group. The responses of the conjunctiva, cornea, and iris to the inoculum were examined and recorded 24, 48, and 72 h after treatment, with recovery observed on Day 3 and 7.

### 2.3 Data processing and analysis

Data were expressed as the mean ± SD and analyzed using GraphPad Prism 7. Data were analyzed for normal distributions using Kolmogorov–Smirnov and Levene’ s tests. If variance was unevenly distributed, equivalent non-parametric tests (primarily Kruskal–Wallis analysis of variance) were used. A *p* < 0.05 value was considered significant.

## 3 Results

### 3.1 Results of the ovicidal activity test

**Figure 2:**
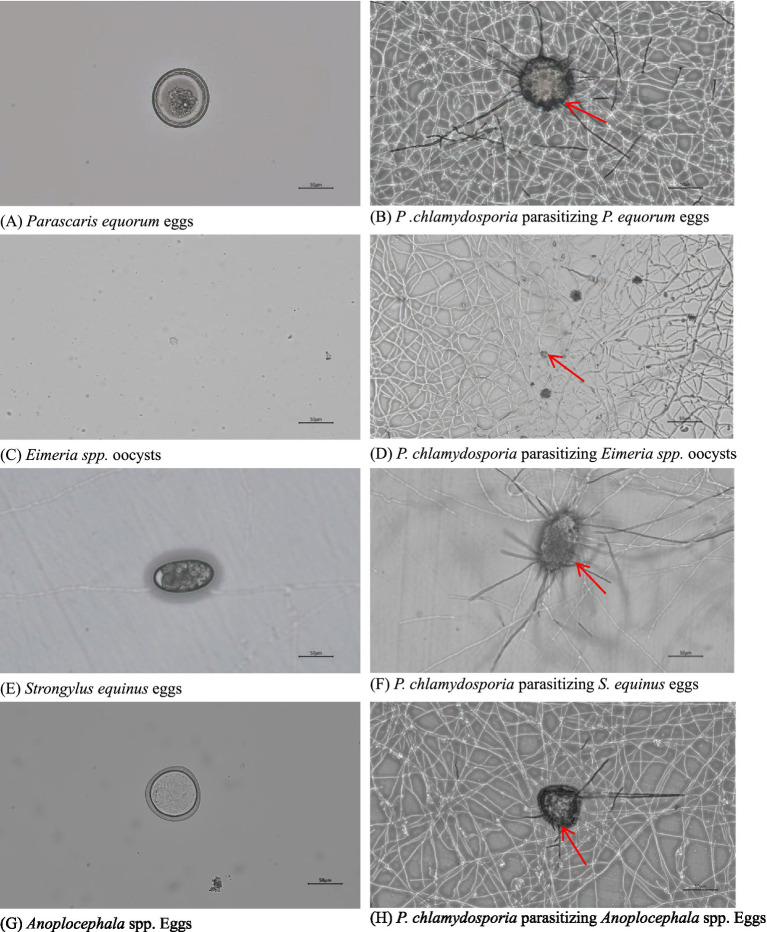
Images of eggs of **(A)** *S. equinus*, **(C)** *P. equorum*, **(E)** *Eimeria* spp., and **(G)** *Anoplocephala* spp. and their parasitism by *P. chlamydosporia* for 7d **(B,D,F,H)**.

Observations showed that *P. chlamydosporia* mycelia first aggregated near the eggs or oocysts, with numerous mycelia then penetrating the egg membrane before gradually destroying the internal structure. The mycelia enter the eggs or oocysts via mechanical and enzymatic actions, leading to significant structural changes that eventually deform the eggs or oocysts, causing their death. *P. chlamydosporia* was found to infect eggs of *S. equinus*, *P. equorum*, *Anoplocephala* spp. and *Eimeria* spp. oocysts, but at different rates, being slower to infect those of *Eimeria* spp. oocysts and *P. equorum* eggs (Table 1 and Figure 2).

**Table 1:** Results of infestation test of *P. chlamydosporia* on eggs of *S. equinus*, *P. equorum*, *Anoplocephala* spp. and *Eimeria* spp. oocysts.

Species	Percentage of eggs or oocysts damaged (%)
*Parascaris equorum*	87.12 ± 7.21
*Eimeria* spp.	47.23 ± 1.52
*Strongylus equinus*	41.25 ± 3.75
*Anoplocephala* spp.	76.32 ± 2.32

### 3.2 Results of acute transoral toxicity test in mice

**Figure 3:**
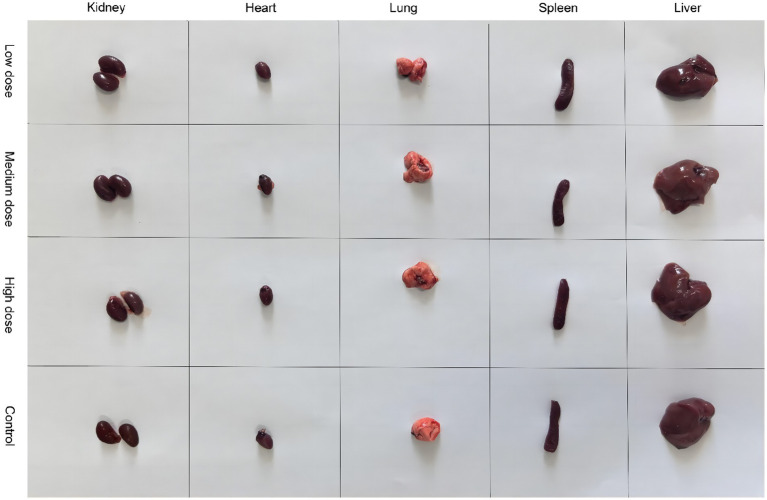
Comparison of the morphology of kidneys, heart, lungs, spleen, and liver from mice in the experimental and control groups.

**Figure 4:**
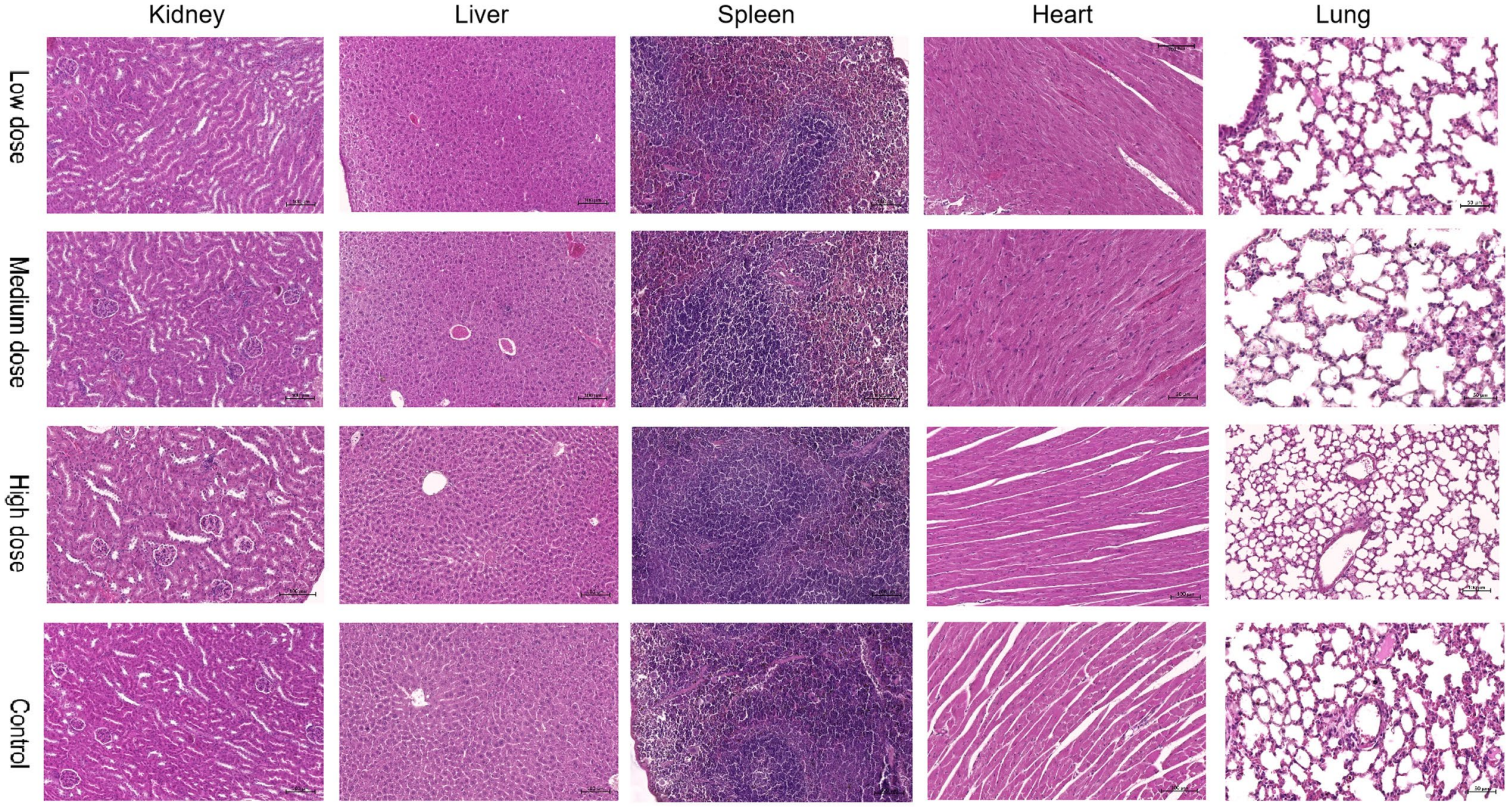
Comparison of pathological images of the kidney, liver, spleen, heart, and lung from mice in the experimental and control groups.

No abnormal clinical observation such as respiration, mental state, appetite, or feces of the experimental or control mice, was made during the test period. The body temperature of each experimental group did not change significantly over the course of the study (Table 2). The b.w. of the mice in each group increased over the course of the study but the changes were not statistically significant (Table 3). There was no significant difference in organ coefficients between the experimental groups (Table 4). Upon autopsy of the mice in the test groups and the control group, no obvious effusion or abnormal content was observed in the thoracic and abdominal cavities, and the location, appearance, and morphology of the tissues and organs were normal; neither was there any change such as dislocation, adhesion, torsion and rupture. All organs were of normal size and texture, with no hemorrhage, scarring, nodular or necrotic change in overall appearance or tissue structure (Figures 3, 4). All blood routine indices and blood biochemical indices were within the normal range in each dose group before and at Day 14 after treatment. There was no significant difference between the groups (Tables 5, 6). The results of the acute oral toxicity test in mice showed that none of the animals had any obvious symptom of poisoning and no death was recorded; LD_50_ was >1 × 10^7^ chlamydospores/kg b.w., which indicated that the acute oral toxicity was of “low toxicity” level.

**Table 2:** Effects of *P. chlamydosporia* inoculum on body temperature in mice (mean ± SD, °C).

Experimental group	Predose	3 days	7 days	14 days
1 × 10^6^/kg	39.50 ± 0.28^a^	39.60 ± 0.29^b^	39.50 ± 0.16^c^	39.60 ± 0.22^d^
5 × 10^6^/kg	39.60 ± 0.28^a^	39.50 ± 0.29^b^	39.49 ± 0.16^c^	39.55 ± 0.22^d^
1 × 10^7^/kg	39.40 ± 0.22^a^	39.53 ± 0.25^b^	39.53 ± 0.19^c^	39.50 ± 0.09^d^
Control	39.43 ± 0.21^a^	39.20 ± 0.29^b^	39.40 ± 0.22^c^	39.46 ± 0.12^d^

Perform a difference comparison between the control group and the high-, medium-and low-dose groups. Different letters in the same column represent significant differences (*p* < 0.05), whereas the same letters represent non-significant differences (*p* > 0.05).

**Table 3:** Effect of *P. chlamydosporia* inoculum on body weight of mice (mean ± SD, °C).

Experimental group	Predose	7 days	14 days
1 × 10^6^/kg	18.73 ± 0.72^a^	21.64 ± 2.08^b^	27.09 ± 2.45^c^
5 × 10^6^/kg	18.57 ± 14.00^a^	22.30 ± 2.11^b^	27.36 ± 2.17^c^
1 × 10^7^/kg	19.29 ± 1.37^a^	23.08 ± 0.55^b^	26.88 ± 1.39^c^
Control	17.29 ± 0.37^a^	22.16 ± 1.35^b^	28.56 ± 1.67^c^

Perform a difference comparison between the control group and the high-, medium-and low-dose groups. Different letters in the same column represent significant differences (*P* < 0.05), whereas the same letters represent non-significant differences (*p* > 0.05).

**Table 4:** Effect of *P. chlamydosporia* inoculum on mouse organ coefficients (X ± SD, %).

Experimental group	Organ coefficient (%)				
	Kidney	Spleen	Heart	Lung	Liver
1 × 10^6^/kg	5.73 ± 0.59^a^	4.54 ± 0.85^b^	5.87 ± 1.35^c^	5.22 ± 0.97^d^	10.25 ± 2.56^e^
5 × 10^6^/kg	5.90 ± 0.46^a^	4.14 ± 0.70^b^	6.21 ± 1.69^c^	5.40 ± 1.97^d^	10.14 ± 3.56^e^
1 × 10^7^/kg	6.49 ± 0.32^a^	4.97 ± 0.94^b^	6.50 ± 2.01^c^	6.79 ± 2.01^d^	11.81 ± 3.11^e^
Control	6.53 ± 0.61^a^	3.83 ± 0.68^b^	6.71 ± 1.50^c^	6.03 ± 0.93^d^	12.32 ± 3.56^e^

Perform a difference comparison between the control group and the high-, medium-and low-dose groups. Different letters in the same column represent significant differences (*P* < 0.05), whereas the same letters represent non-significant differences (*P* > 0.05).

**Table 5:** Effects of *P. chlamydosporia* administration on blood routine indices in mice (mean ± SD).

Index	Control	1 × 10^6^/kg	5 × 10^6^/kg	1 × 10^7^/kg	Normal range
WBC	4.21 ± 1.99^a^	4.55 ± 2.36^a^	4.78 ± 1.25^a^	5.60 ± 1.95^a^	0.8–6.8
Lymph	3.25 ± 1.74^b^	3.60 ± 2.05^b^	3.43 ± 1.04^b^	4.20 ± 1.67^b^	0.7–5.7
Mon	0.13 ± 0.05^c^	0.13 ± 0.05^c^	0.16 ± 0.08^c^	0.16 ± 0.05^c^	0.0–0.3
Gran	0.83 ± 0.30^d^	0.81 ± 0.34^d^	1.18 ± 0.45^d^	1.23 ± 0.40^d^	0.1–1.8
Lymph%	76.01 ± 6.29^e^	76.13 ± 8.00^e^	71.36 ± 8.12^e^	73.73 ± 6.03^e^	55.8–90.6
Mon%	2.90 ± 0.79^f^	3.65 ± 1.65^f^	3.71 ± 1.08^f^	3.53 ± 0.40^f^	1.8–6.0
Gran%	21.08 ± 5.66^g^	20.21 ± 6.48^g^	24.91 ± 7.29^g^	22.73 ± 5.75^g^	8.6–38.9
RBC	8.79 ± 1.55^h^	8.46 ± 1.15^h^	7.79 ± 1.83^h^	9.07 ± 1.13^h^	9.36–9.42
HGB	139.00 ± 25.45^i^	137.33 ± 24.76^i^	131.00 ± 30.82^i^	152.66 ± 30.08^i^	110–143
HCT	41.70 ± 6.95^j^	42.11 ± 7.61^j^	39.98 ± 9.40^j^	46.06 ± 9.00^j^	34.6–44.6
MCV	47.56 ± 1.82^k^	49.55 ± 2.44^k^	51.31 ± 0.89^k^	50.53 ± 3.92^k^	48.2–58.3
MCH	15.76 ± 0.72^l^	16.06 ± 0.81^l^	16.75 ± 0.25^l^	16.66 ± 1.38^l^	15.8–19
MCHC	332.33 ± 8.59^m^	325.50 ± 2.73^m^	327.16 ± 3.18^m^	330.66 ± 5.68^m^	302–353
RDW	14.80 ± 1.15^n^	14.86 ± 1.08^n^	15.10 ± 1.09^n^	15.03 ± 0.40^n^	13–17
PLT	559.50 ± 307.77^o^	864.66 ± 508.69^o^	712.80 ± 279.84^o^	792.33 ± 435.91^o^	450–1,590
MPV	5.38 ± 0.30^p^	5.60 ± 0.38^p^	5.86 ± 0.35^p^	5.96 ± 0.15^p^	3.8–6
PDW	16.16 ± 0.30^q^	16.20 ± 0.08^q^	16.16 ± 0.20^q^	16.40 ± 0.20^q^	-
PCT	0.29 ± 0.14^r^	0.32 ± 0.20^r^	0.42 ± 0.17^r^	0.33 ± 0.14^r^	-

Perform a difference comparison between the control group and the high-, medium-and low-dose groups. Different letters in the same column represent significant differences (*P* < 0.05), whereas the same letters represent non-significant differences (*P* > 0.05).

**Table 6:** Effects of *P. chlamydosporia* administration on mouse blood biochemical indices (mean ± SD).

Index	Control	1 × 10^6^/kg	5 × 10^6^/kg	1 × 10^7^/kg	Normal range
ALB	35.06 ± 2.92^a^	33.96 ± 2.59^a^	34.33 ± 4.21^a^	36 ± 3.55^a^	20–48
TP	38.50 ± 14.78^b^	39.80 ± 11.27^b^	41.36 ± 2.379^b^	35.36 ± 6.49^b^	36–66
GLOB	33.43 ± 12.01^c^	35.84 ± 8.90^c^	31.03 ± 18.24^c^	29.36 ± 2.96^c^	-
A/G	1.11 ± 0.26^d^	0.97 ± 0.16^d^	0.91 ± 0.24^d^	1.23 ± 0.02^d^	-
TB	0.13 ± 0.05^e^	1.16 ± 1.07^e^	0.33 ± 0.40^e^	0.60 ± 0.86^e^	0–15
AST	64.01 ± 57.10^f^	246.40 ± 115.41^f^	81.33 ± 66.14^f^	161.00 ± 30.41^f^	59–247
ALT	130.67 ± 173.66^h^	57.80 ± 13.33^h^	67.33 ± 19.63^h^	45.00 ± 17.52^h^	28–132
AMY	2174.20 ± 192.75^i^	2392.40 ± 266.91^i^	2438.00 ± 84.53^i^	2469.00 ± 210.38^i^	1691.00–3,615
CK	577.67 ± 502.23^j^	1632.40 ± 1233.47^j^	363.66 ± 67.24^j^	720.67 ± 52.14^j^	68–1,070
Crea	29.10 ± 12.86^k^	25.92 ± 8.73^k^	30.00 ± 9.20^k^	27.13 ± 8.14^k^	12–71
BUN	10.36 ± 5.55^l^	10.36 ± 2.14^l^	9.93 ± 4.08^l^	9.33 ± 1.41^l^	4–11.8
GLU	4.18 ± 2.93^m^	4.68 ± 3.89^m^	4.32 ± 3.07^m^	6.71 ± 0.63^m^	5–10.67
TG	1.20 ± 0.09^n^	0.93 ± 0.41^n^	1.12 ± 0.71^n^	1.26 ± 0.18^n^	0.62–1.63
Ca	2.40 ± 0.17^o^	2.43 ± 0.18^o^	2.43 ± 0.03^o^	2.60 ± 0.11^o^	1.48–2.35
PHOS	2.22 ± 0.03^p^	2.99 ± 0.11^p^	2.89 ± 0.65^p^	2.51 ± 0.12^p^	1.97–3.26

Perform a difference comparison between the control group and the high-, medium-and low-dose groups. Different letters in the same column represent significant differences (*P* < 0.05), whereas the same letters represent non-significant differences (*P* > 0.05).

#### 3.2.1 Results of the acute skin irritation test

The results of the skin sensitization test showed that no erythema or edema formed in the skin of the treated mice. The results of the acute single skin irritation test were negative, which indicated that the *P. chlamydosporia* inoculum was “non-irritating” in terms of this single skin irritation test (Table 7).

**Table 7:** Skin reactions.

Eryformation (score)	score			Edema formation (score)	Score		
	24 h	48 h	72 h		24 h	48 h	72 h
No erythema (0)	0	0	0	No edema (0)	0	0	0
Mild erythema (1)	–	–	–	Mild edema, barely visible (1)	–	–	–
Moderate erythema (2)	–	–	–	Moderate edema, and clearly visible (2)	–	–	–
Severe erythema (3)	–	–	–	Severe edema, skin bulge of 1 mm and clear outline (3)	–	–	–
Purple red erythema to mild eschar formation (4)	–	–	–	Severe edema, skin bulge of >1 mm or blisters or ulceration (4)	–	–	–

#### 3.2.2 Results of eye irritation test

The results of the eye irritation test in mice showed no edema or secretion, and no abnormality of cornea and iris, indicating that the *P. chlamydosporia* inoculum was not irritating to the mice.

## 4 Discussion

Biological control using *P. chlamydosporia* is an environmentally friendly control method to support the use of sustainable agriculture and reduce chemical pesticide use. *P. chlamydosporia* is specialized in attacking the eggs, females, and cysts of animal and plant parasites (14). The use of this type of bioprophylactic fungi for the control of parasites in livestock is widely supported because it leaves no drug residues, the parasites do not develop drug resistance, it does not contaminate the natural environment, and it kills the eggs in feces and the environment before they hatch and infect livestock; such effects greatly advance the control of parasites and pose no harm to livestock (15, 16). So far, *P. chlamydosporia* strains have been isolated and screened in Brazil, Poland, the UK, Australia and Sweden (17–19) and have been shown to be adapted to, and showed better action when in their natural habitats; researchers have investigated their role in controlling the eggs of *Ascaris suum*, *Ascaridia galli*, and *Toxocara canis*, showing nematicidal efficiencies of up to 94.8% (16, 20–22). The main virulence factors of *P. chlamydosporia* are serine proteases, chitinases, and various toxins (23, 24). Their mechanism of action is based on a mechanical/enzymatic process, which enables the penetration of their hyphae into helminth eggs, with subsequent internal colonization of these. Thus, morphological changes occur in the eggshell, as well as in the embryo, making it nonviable (10). Nematode infestation by this fungus involves the recognition of females and eggs by the fungus, the subsequent degradation of nematode eggshells by the fungus, and finally, the invasion and dissipation of nematodes by the mycelia (25). Although the ovicidal activity of *P. chlamydosporia* has been frequently evaluated, it is not known whether it has destructive power against larvae. Vieira et al. specifically studied the ability of *P. chlamydosporia* to capture larvae and found that it reduced 66.8% of L3 GINs in cattle, but there are few reports in the literature on the larvicidal activity of this fungus (26, 27). Among these degradative enzymes, serine protease and chitinase are key virulence factors in the infestation and lethality of nematodes (28, 29). The extracellular serine protease VCP1 degrades the outer proteins of the eggshell and exposes the inner chitin layer (30).

Although *P. chlamydosporia* has shown potential for clinical applications, there has been a lack of data regarding its efficacy against equine parasitic eggs and oocysts. Braga et al. (10) reported that a crude enzymatic extract from *P. chlamydosporia* (VC4) reduced the hatching of cyathostomin eggs, while Braga et al. (31) suggested *P. chlamydosporia* to be an effective biological control agent of *O. equi* eggs in natural conditions. The results of Braga et al. indicate that P. chlamydosporia can be used in the control of eggs of gastrointestinal helminth parasites that hatch in a short period of time in the environment. Before this, only helminth eggs with long periods of development in the environment have been studied (14). In our study, regardless of the length of the development time in the environment, the fungus exhibited ovicidal activity against different worm eggs and oocysts after being co-cultured for 7 days. However, there were differences in its effectiveness. Braga et al. also demonstrated that *P. chlamydosporia* exhibited ovicidal activity on equine eggs following passage through the gastrointestinal tract. The efficacy of this fungus was enhanced when used in conjunction with the nematode-feeding fungus *Paecilomyces lilacinus* [belongs to *Purpureocillium*, (40)] which has a distinct mechanism of action (31). Furthermore, the fungus *Duddingtonia flagrans* also acts as the main predatory fungus of gastrointestinal parasitic nematode larvae. Combined administration of *D. flagrans* and *P. chlamydosporia* fungal products reduced the presence of eggs and larvae in pastures, indicating their effectiveness in the strategic control of gastrointestinal parasites in cattle. Another species capable of adhering to the surface of the eggs of certain helminths, penetrating and feeding on their contents, is Mucor circinelloides. It is a filamentous saprophytic fungus with action against trematode eggs (*F. hepatica*, *Calicophoron daubneyi*), ascarids (*Toxocara canis*, *Toxascaris leonina*, *A. suum*, *Bayliscascaris procyonis*), and whipworms (*Trichuris* spp.) (32). The current results showed that *P. chlamydosporia* destroyed the *S. equinus* eggs, *Eimeria* spp. oocysts, and *Anoplocephala* spp. eggs, although their effect on *P. equorum* was not as effective, mainly because of the fast hatching speed of this parasite. In addition, the mycelia of *P. chlamydosporia* demonstrated a tendency to aggregate close to the parasitic eggs. This phenomenon might be associated with the release of substances by the eggs, which could influence the growth of the mycelia, which then penetrate, and destroy, the eggs (33). Other previous reports indicated the possibility of some eggshell components (such as chitin) acting as stimuli for the fungi can identify them and develop hyphae against them (23, 34).

The current study also used a mouse model to assess the safety of *P. chlamydosporia* as a biological agent in terms of any possible effects on nontarget organisms. Such studies are vital for the protection of human health and the environment, as well as for the successful commercialization of this fungus as a biopesticide. The lyophilized formulations of *P. chlamydosporia* at different doses did not produce any toxicity in mice and neither was there any buildup in the feces of the treated mice over the 14-day test period, indicating that solutions of *P. chlamydosporia* could be safe for use in livestock. In addition, microbiological analysis of mouse feces after 14 days of treatment demonstrated that no conidia of the fungus were identified. This suggested that *P. chlamydosporia* did not undergo a multiplicative buildup in the body.

The plant parasite killer Rizotec^®^, which contains *P. chlamydosporia*, has become an alternative solution for controlling plant parasitic nematodes, showing high parasitism efficiency on eggs, control of juvenile and adult female nematodes, and resulting in a reduction in the number of eggs and female nematodes in sugarcane fields. Studies have investigated the toxicity and ecotoxicity of Rizotec on animals including rats, rabbits, and quail (35, 36), and invertebrates (37), finding no toxic, irritant, pathogenic, or infectious effects, indicating that this compound is safe to use, in agreement with the current study.

Thus, the results of the current study provided a theoretical basis for understanding the process of parasite egg infection by *P. chlamydosporia* and promoting its application as a biological control agent for the control of gastrointestinal parasites in animals. *P. chlamydosporia* showed good oocidal effects against the equine parasites tested and had no toxic effects on mice; thus, we suggest that it would be safe to use in livestock.

## Data Availability

The original contributions presented in the study are included in the article/supplementary material, further inquiries can be directed to the corresponding authors.
